# T Cell Immunity Evaluation and Immunodominant Epitope T Cell Receptor Identification of Severe Acute Respiratory Syndrome Coronavirus 2 Spike Glycoprotein in COVID-19 Convalescent Patients

**DOI:** 10.3389/fcell.2021.696662

**Published:** 2021-11-03

**Authors:** Luo Li, Qian Chen, Xiaojian Han, Meiying Shen, Chao Hu, Siyin Chen, Jing Zhang, Yingming Wang, Tingting Li, Jingjing Huang, Shenglong Li, Yanan Hao, Aishun Jin

**Affiliations:** ^1^Department of Immunology, College of Basic Medicine, Chongqing Medical University, Chongqing, China; ^2^Chongqing Key Laboratory of Cancer Immunology Translational Medicine, Chongqing Medical University, Chongqing, China; ^3^Department of Endocrine Breast Surgery, The First Affiliated Hospital of Chongqing Medical University, Chongqing, China

**Keywords:** SARS-CoV-2, COVID-19, T cell epitope, TCR, TCR-T

## Abstract

A better understanding of the role of T cells in the immune response to Severe Acute Respiratory Syndrome Coronavirus 2 (SARS-CoV-2) is helpful not only for vaccine development but also for the treatment of COVID-19 patients. In this study, we determined the existence of SARS-CoV-2-specific T cells in the blood of COVID-19 convalescents. Meanwhile, the specific T cell response in the non-RBD region was stronger than in the RBD region. We also found that SARS-CoV-2 S-specific reactive CD4^+^ T cells exhibited higher frequency than CD8^+^ T cells in recovered COVID-19 patients, with greater number of corresponding epitopes presented. Importantly, we isolated the SARS-CoV-2-specific CD4^+^ T cell receptors (TCRs) and inserted the TCRs into allogenic CD4^+^ T cells. These TCR-T cells can be activated by SARS-CoV-2 spike peptide and produce IFN-γ *in vitro*. These results might provide valuable information for the development of vaccines and new therapies against COVID-19.

## Introduction

Severe acute respiratory syndrome coronavirus 2 (SARS-CoV-2) has been identified as the cause of coronavirus disease 2019 (COVID-19). As of June 17, 2021, more than 3 million deaths and 176 million cases have been reported worldwide.^1^ Vaccines play an important role in the battle against COVID-19, and multiple vaccines have been rolled out in countries (Anderson et al., 2020; Polack et al., 2020; Voysey et al., 2021; Xia et al., 2021). The level of neutralizing antibody has been reported to decrease both in most of the asymptomatic and symptomatic groups during the early convalescent phase (Long et al., 2020), with no high concentration of neutralizing activity found even in most recovered plasma samples (Li et al., 2020; Robbiani et al., 2020), suggesting that the effective window for the neutralizing antibody induced by vaccines may be relatively limited.

According to the research of severe acute respiratory syndrome coronavirus (SARS-CoV) and Middle East respiratory syndrome coronavirus (MERS-CoV), T cell immunity plays a decisive part in the disease recovery and long-lasting protection (Zhao et al., 2010, 2016; Channappanavar et al., 2014a; Ng et al., 2016). T cells can clear the virus even in the absence of antibodies and when the innate immune system is not activated (Zhao et al., 2010). Meanwhile, the depletion of CD4^+^ T cells during SARS-CoV infection will delay virus clearance (Chen et al., 2010). Furthermore, T cells help clean the SARS-CoV and, at the same time, form a long-lasting memory response to it (Fan et al., 2009; Ng et al., 2016). SARS-CoV-2 can induce virus-specific T cells in the absence of seroconversion (Sekine et al., 2020; Gallais et al., 2021). Hence, for SARS-CoV-2, T cell-mediated cellular immunity may present certain beneficial advances over antibodies, and a strong and extensive T cell response may be essential for continuous immune protection against SARS-CoV-2. T cell-mediated cellular immunity largely depends on the recognition of its antigen presented by the major histocompatibility complex (MHC) through its T cell receptors (TCRs; Hu et al., 2021). Therefore, it is crucial to obtain SARS-CoV-2-specific TCRs with high functionalities for the immunological protection of this highly mutated coronavirus.

The current study aimed to investigate the T cells’ response to SARS-CoV-2 spike protein (S protein) and identify the S-specific TCR. We found a stronger and broader SARS-CoV-2 S protein-specific CD4^+^ T cell response in most COVID-19 convalescent patients. Specifically, we have identified an immunodominant epitope B65 within the S protein, which presents a unique value for the peptide vaccine development. Further, we found an S-specific TCR targeting the immunodominant peptide B65. We also showed that normal CD4^+^ T cells constructed with B65-targeted TCRs exhibit functional reactivity against SARS-CoV-2 S. Our study provided valuable information for developing potential vaccines and new treatment strategies against COVID-19.

## Materials and Methods

### COVID-19 Convalescent Patients and Healthy People

Blood samples of COVID-19 convalescent patients were collected from the Yongchuan Hospital of Chongqing Medical University and the Third Affiliated Hospital of Chongqing Medical University. Healthy people’s blood samples, which were never exposed to SARS-CoV-2 or chronic infections, include hepatitis B or C, HIV, and Syphilis, which were collected from the Center of Immunology Research, Chongqing Medical University. This project was approved by the ethics committee of Chongqing Medical University (20200310). Informed consent was obtained from all individual donors before this research.

### Peripheral Blood Mononuclear Cell Isolation

Whole blood was diluted with an equal volume of Dulbecco’s phosphate-buffered saline (PBS) with 2% fetal bovine serum (FBS, Gibco) and mixed gently. The diluted samples were pipetted down the side of the SepMate^TM^-50 tube (Stemcell Technologies, Vancouver, BC, Canada) with added Lymphoprep^TM^ (Stemcell). To separate peripheral blood mononuclear cells (PBMCs), the diluted blood samples with density gradient medium were centrifuged at 1,200 × *g* for 10 min with the brake applied at room temperature. PBMCs were washed twice using PBS supplemented with 2% FBS (between the washes, spinning at 1,000 g, for 7 min at RT, and discarding the supernatant) and were cryopreserved until subsequent analysis.

### Enrichment of IFN-γ-Secreting and CD4^+^T Cells

For the enrichment of IFN-γ-secreting T cells, peptide pools (5 μM/peptide) for 6 h were used to stimulate the PBMCs of recovered COVID-19 patients. Then, IFN-γ-secreting T cells were caught using IFN-γ Secretion Assay Cell Enrichment and Detection Kit (Miltenyi Biotec, Bergisch Gladbach, Germany). CD4^+^T cells were selected by EasySep Human CD4^+^ T Cell Enrichment Kit (Stemcell).

### T Cell Receptor Sequencing and Analysis

T cell receptor sequencing and analysis were performed as previously described (Hu et al., 2021). In short, RT PCR and nested PCR were used to amplify the TCRα and TCRβ chain genes. Then, the PCR products were analyzed by sequencing (Tsingke, Guangzhou, China). The IMGT/V-QUEST tool was used to analyze the TCR repertoire.

### Cell Culture

Peripheral blood mononuclear cells and CD4^+^Jurkat cells were cultured in RPMI 1640 (Gibco) containing 10% FBS, 2 mM GlutaMAX^TM^, 25 mM HEPES, and 10 μg/ml gentamicin (Gibco, Grand Island, NY, United States) and 55 μM 2-mercaptoethanol (Sigma-Aldrich, St. Louis, MO, United States). 293T cells were cultured in Dulbecco’s modified Eagle’s medium (DMEM, Gibco) supplemented with 10% FBS, 2 mM GlutaMAX^TM^, 1 mM sodium pyruvate (Gibco), and MEM NEAA supplement (Gibco). IFN-γ-secreting T cells were expanded using irradiated (50-Gy) allogeneic PBMCs at a ratio of 1:100. The cells were cultured in a mixture medium [complete media: stocktickerAIM-V media (Gibco) = 1:1] containing 30 ng/ml OKT3 antibody (Miltenyi Biotec) and 3,000 IU/ml IL2 (PeproTech). All cells were cultured for 14 days before use.

### Construction of Lentivirus Vectors and Cell Infection

Lentivirus vector construction and lentivirus production were established as previously described (Hu et al., 2021). To generate TCR-transduced-CD4^+^ T and TCR-transduced-CD4^+^ Jurkat cells, CD4^+^ T cells were stimulated with 1 μg/ml OKT3 antibody and 1 μg/ml anti-CD28 antibody (Miltenyi Biotec) and cultured in a complete medium containing 100 IU/ml IL-2 for 48 h. CD4^+^ Jurkat cells and stimulated CD4^+^ T cells were seeded into 24-well-plates and incubated with 500 μl lentivirus and 500 μl RPMI 1640 for 6 h. Next, 500 μl complete medium was added to each well. After 24 h, the original medium was replaced with a 2-ml complete medium and incubated for 48 h. Then, the cells were harvested and used for subsequent assays.

### Stimulation of Peripheral Blood Mononuclear Cells With Peptides and Cocultured Experiment

Peripheral blood mononuclear cells were stimulated with peptide (5 μM) 24 or 48 h after resting overnight in complete medium. Supernatants of 24 h were collected for enzyme-linked immunosorbent assay (ELISA) to detect cytokine. Stimulated cells of 48 h were used to test SARS-CoV-2 specific T cells by flow cytometry. For a cocultured experiment: PBMCs as presenting cells were labeled with a 5-μM peptide for 2 h at 37°C. The PBMCs were washed twice with PBS before coculturing with TCR-transduced CD4^+^ T or TCR-transduced-CD4^+^ Jurkat cells for 24 or 6 h, respectively. Flow cytometry was used to check the activated cells.

### Flow Cytometry Analysis

The immunological phenotypes of PBMCs were assessed by flow cytometry using the following surface markers: BV510-anti-CD3, PerCP-Cy5.5-anti-CD4, FITC-anti-CD8, PE-anti-CD19, APC-anti-CD45RA, BV412-anti-CCR7, BV605-anti-CD56, PE-anti-CD14, APC-anti-CD16 (BioLegend). The IFN-γ-secreting T cells were detected using PE-anti-IFN-γ (Miltenyi Biotec) and BV510-anti-CD3. After stimulation with peptide for 48 h, the SARS-CoV-2 specific T cells were tested applying BV510-anti-CD3, PerCP-Cy5.5-anti-CD4, FITC-anti-CD8 and BV421-anti-CD137, and APC-anti-CD134 (BioLegend, San Diego, CA, United States). The activations of TCR-transduced-CD4^+^ Jurkat and TCR-transduced-CD4^+^ T cells were assayed using PerCP-Cy5.5-anti-CD4, APC-anti-mTCRβ, PE-anti-CD69, PE-anti-IFN-γ, and BV421-anti-CD137. In brief, cells were stained with various Abs at room temperature for 20 min. Then, the cells were washed with PBS and stained for 15 min using LIVE/DEAD viability dye (Thermo Fisher Scientific, Waltham, MA, United States). Data were acquired using the FACSCelesta cytometer and analyzed by FlowJo software. For single-cell acquisition, CD3^+^CD4^+^CD134^+^CD137^+^T cells were sorted into 96-well PCR plates (Bio-Rad) at a single-cell level using FACSAria III.

### IFN-γ ELISA Assays

IFN-γ capture antibody (2 μg/ml, BioLegend) and IFN-γ detection antibody (1 μg/ml, BioLegend) were used to detect cytokine. The ELISA plate (Corning) was coated overnight with a capture antibody at 4°C and washed three times with PBS containing 0.05% Tween 20 (PBST), then blocked with 3% BSA at 37°C for 1 h. Then, supernatants were added to the ELISA plate (50 μl/well) and incubated at 37°C for 1 h. After washing with PBST for five times, the detection antibody was added and incubated at 37°C for 30 min. The plate was incubated with streptavidin-ALP (1:1,000) at 37°C for 30 min after washing with PBST five times. Next, the plate was washed with PBST six times, followed by incubation with 50 μl pNPP solution (Mabtech, Stockholm, Sweden) at 37°C for 40 min. Finally, the plate was analyzed *via* the Varioskan LUX Multimode Microplate Reader at 405 nm.

### Statistical Analysis

GraphPad Prism 8.0 was used for statistical analyses. Data are shown as the mean ± SD. The unpaired *t*-test and Spearman’s test were used for data analysis. *p* < 0.05 was considered statistically significant.

## Results

### Immunological Phenotypes of Recovered COVID-19 Patients

Peripheral blood samples from 10 recovered individuals of COVID-19 were collected to study the T cells’ immune response to SARS-CoV-2. All participants were diagnosed with mild COVID-19, including four men and six women, ranging from 32 to 59 years old, with a median age of 47. From symptom onset or positive PCR results to discharge, the recovery time is 7–26 days, with a median recovery time of 18 days. As a control group, we collected 10 healthy people who had not been infected with SARS-CoV-2.

**FIGURE 1:**
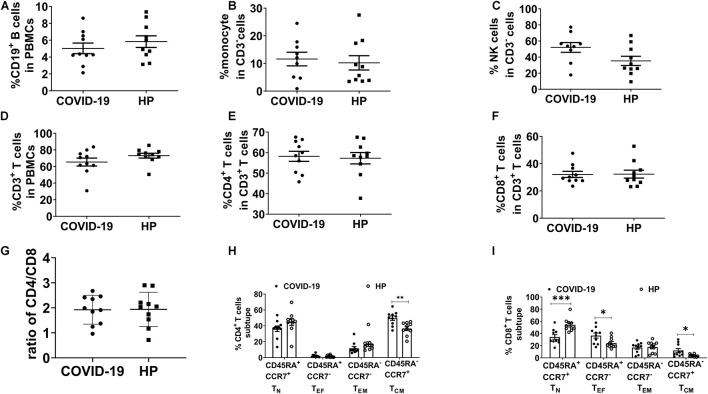
Immunophenotyping of PBMC in recovered COVID-19 patients and HP. Multicolor FC was used to detect the frequency of **(A)** B cells (CD3^–^CD19^+^), **(B)** monocytes (CD3^–^CD16^–^CD14^+^), **(C)** NK cells (CD3^–^CD56^+^CD16^+^), **(D)** CD3^+^ T cells, **(E)** CD4^+^ T cells (CD3^+^CD4^+^CD8^–^), and **(F)** CD8^+^ T cells (CD3^+^CD4^–^CD8^+^). **(G)** The ratio of CD4/CD8. **(H,I)** T_CM_ (CD45RA^–^CCR7^+^), T_N_ (CD45RA^+^CCR7^+^), T_EF_ (CD45RA^+^CCR7^–^), and T_EM_ (CD45RA^–^CCR7^–^) in 10 recovered COVID-19 patients and 10 HP. FC: flow cytometry; PBMCs: peripheral blood mononuclear cells; COVID-19: Coronavirus Disease-2019; HP, healthy people; T_CM_, central memory T cells; T_N_, naïve T cells; T_EF_, effector T cells; T_EM_, effector T memory cells. *P < 0.05, **P < 0.01, ***P < 0.001.

To characterize cellular immunological phenotypes, we isolated PBMCs from the convalescent patients and the healthy controls for subsequent multicolor flow cytometry analysis. The proportions of B cells, monocytes, and natural killer (NK) cells were similar in the two groups (Figures 1A–C). Meanwhile, no significant differences were observed in the frequencies of CD3^+^, CD4^+^, and CD8^+^ T cells between the recovered group and the control group (Figures 1D–G). We also assess the subtype of CD4^+^ and CD8^+^ T cells. The frequencies of CD4^+^ central memory T cells (T_CM_), CD8^+^ T_CM_, and CD8^+^ effector T cells (T_EF_) were relatively higher in the recovered group than in the control group (Figures 1H,I).

### Severe Acute Respiratory Syndrome Coronavirus 2 Spike Glycoprotein-Reactive T Cells in Recovered COVID-19 Patients

**FIGURE 2:**
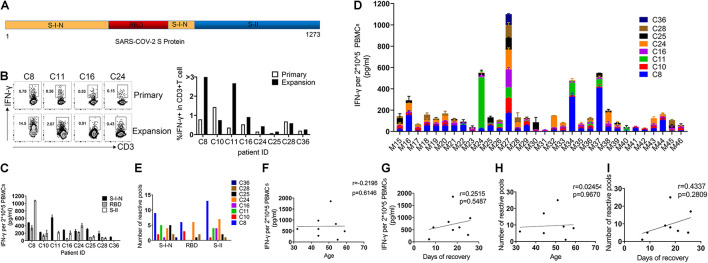
Severe Acute Respiratory Syndrome Coronavirus 2 spike glycoprotein-reactive T cells in recovered COVID-19 patients. **(A)** The structural of SARS-COV-2 spike glycoprotein: the N-terminal of S protein is called S-I, including RBD and non-RBD regions (S-I-N), and the C-terminal of the S protein is called S-II. **(B)** FC was used to detect the frequency of SARS-CoV-2 S-specific T cells (CD3^+^IFN-γ^+^) before and after expansion. **(C)** The IFN-γ production in PBMCs of each individual was detected by ELISA after stimulation with SARS-COV-2 S peptides (24 h). A total of 2 × 10^–5^ PBMCs were stimulated with complex Mix pools of different regions of SARS-COV-2 S protein: S-I-N (Mix21-31), RBD (Mix15-20), or S-II (Mix32-46). **(D)** The magnitude of T cell response for each individual to a single Mix pool. **(E)** The breadth of T cell response for each individual to different regions of SARS-COV-2 S protein. The breadth of T cell response was calculated by the number of positive Mix pools. The number of positive mix pools = IFN-γ/DMSO > 2. **(F–I)** The correlation analyses of magnitude and breadth of T cell responses versus patients’ age **(F,H)** and the time of recovery **(G,I)**. The quantitative results are shown as the mean ± SD of at least two independent experiments. SARS-CoV-2, severe acute respiratory syndrome coronavirus 2; RBD, receptor-binding domain; FC, flow cytometry; PBMCs, peripheral blood mononuclear cells; IFN-γ, interferon-γ; ELISA, enzyme linked immunosorbent assay; Mix, mixture.

To study SARS-CoV-2-specific T cell response, we selected S protein for being a key protein for viral entry affecting viral infections and being a target for vaccines (Anderson et al., 2020; Polack et al., 2020; Wrapp et al., 2020; Voysey et al., 2021; Weinreich et al., 2021; Xia et al., 2021). To this end, we designed 317 peptides containing 15 amino acids (aa), with 11-aa overlaps, to span the entire S protein. These peptides were divided into 32 mixed pools (Mix), each consisting of 10 peptides. The N-terminal of the S protein is designated as S-I, containing non-receptor-binding domain (non-RBD, S-I-N; Mix21-31) regions and the RBD (Mix15-20), while the C-terminal of the S protein is called SII (Mix32-46) (Figure 2A). To increase the amount of SARS-CoV-2-specific T cells, we performed rapid expansion *in vitro*. As shown in Figure 2B, the expansion process successfully increased the frequencies of SARS-CoV-2 specific T cells in 75% samples, although with only a slight effect on the samples of C10 and C28. Then, we determined the activation of the specific T cell populations by ELISA for the IFN-γ secretion post *in vitro* restimulation with different peptide pools for 24 h. We found that T cells specifically responding to multiple regions of the S protein do exist in the PBMCs of all recovered COVID-19 patients. These specific T cells demonstrated different reactivities to different regions of SARS-COV-2 S, which were 100% for S-I-N, 75% for RBD, and 87.5% for S-II. Of note, the RBD region accounted for the weakest T cell response from the majority of patient samples, including two that showed barely detectable levels of IFN-γ (Figure 2C). Further, we analyzed the magnitude and breadth of T cell reactivity to different S protein regions in detail. Collected T cell responses to individual Mix were compared, and we found that both S-I-N and S-II corresponded to larger numbers of peptide pools with better potential to induce IFN-γ secretion, despite individual variations (Figures 2D,E). To investigate the clinical relevance of such T cell reactivity, we used Spearman’s linear correlation and found no significant correlation between the magnitude and breadth of T cell responses and patient age or the time of recovery (Figures 2F–I). Overall, these results indicated that the PBMCs of recovered COVID-19 patients contained specific populations of T cells with high reactivity to SARS-CoV-2 S protein, of which the RBD region might induce a relatively lower level of T cell responses.

### Identification of Severe Acute Respiratory Syndrome Coronavirus 2 S Protein-Specific CD4^+^ and CD8^+^ T Cell Epitopes

**FIGURE 3:**
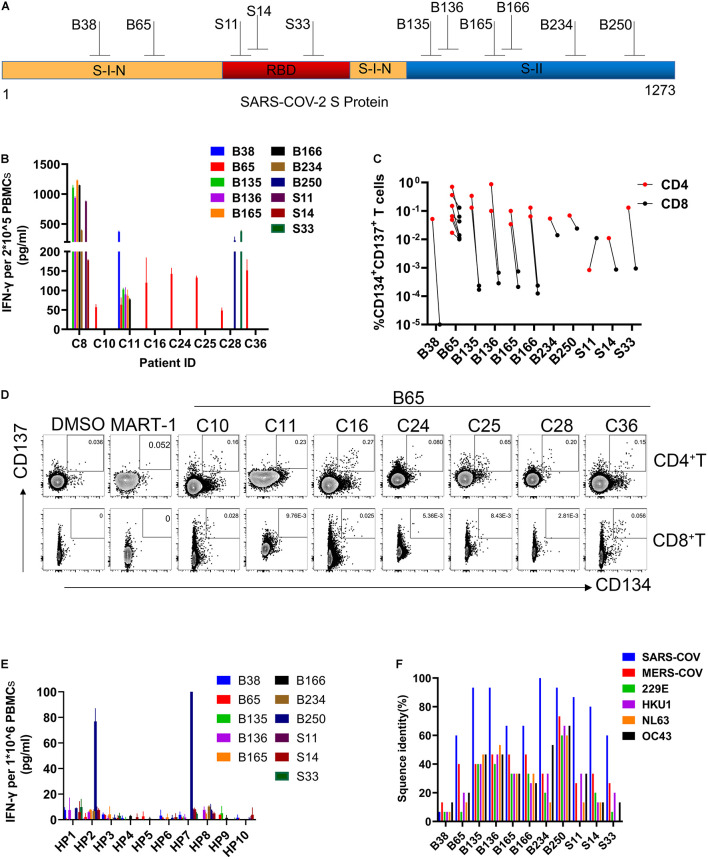
Identification of SARS-CoV-2 S protein-specific CD4^+^ and CD8^+^ T cell epitopes. **(A)** The structure of SARS-COV-2 spike glycoprotein. **(B)** IFN-γ was determined by ELISA. 2 × 10^–5^ PBMCs for each participant were stimulated with a single peptide from positive mix pools (24 h). **(C)** The frequency of reactive CD4 + and CD8 + T cells to each peptide in participants. **(D)** FC plot example. FC was used to detect the reactive CD4 + T cells (CD3 + CD4 + CD8-CD134 + CD137 +) or CD8 + T cells (CD3 + CD4-CD8 + CD137 + CD134 +) in PBMCs stimulated by positive peptide or MART-1 (negative control) (48 h). **(E)** IFN-γ ELISA assays were used to evaluate the cross-reactivity of HP to peptide. 1 × 10^–6^ PBMCs for each HP were stimulated with each peptide (24 h). **(F)** The proportion of sequence identity of SARS-CoV-2 spike glycoprotein to the spike glycoproteins of SARS-COV, MERS-COV, and human common cold coronaviruses 229E, HKU1, NL63, and OC43. IFN-γ, interferon-γ; ELISA, enzyme-linked immunosorbent assay; PBMCs, peripheral blood mononuclear cells; FC, flow cytometry; SARS-CoV-2, severe acute respiratory syndrome coronavirus 2; SARS-COV, severe acute respiratory syndrome coronavirus; MERS-COV, Middle East respiratory syndrome coronavirus (MERS-CoV); MART-1, melanoma-associated antigen.

To better characterize the specific epitopes for SARS-CoV-2 S-reactive T cells, single peptides from each positive mixture pool were used to stimulate T cells from recovered COVID-19 patients. A total of 11 peptides containing SARS-CoV-2 T cell epitopes were recognized by recovered COVID-19 patients PBMCs, 2 from S-I-N, 3 from RBD, and 6 from S-II (Figures 3A,B and Table 1). The T cells from C8 and C11 participants recognized 7 and 6 peptides, respectively (Figure 3B and Table 1). Furthermore, we determined whether these 11 positive peptides had preferential correspondence to CD4^+^ or CD8^+^ T cells. Flow cytometry analysis showed that the frequency of SARS-CoV-2-specific CD4 + T cells was higher than that of CD8^+^ T cells (Figure 3C). Specifically, peptide B65 from S-I-N was recognized in seven out of eight participants (87.5%) and contained at least two epitopes recognized by CD4^+^ T cells or CD8^+^ T cells (Figure 3D and Table 1). Moreover, we found that 10 of 11 peptides induced the activation of CD4^+^ T cells, while only four peptides induced the activation of CD8^+^ T cells (Table 1). Two patients, C8 and C11, whose samples were reactive to more peptides than the rest of the participants, contain both CD4^+^ and CD8^+^ SARS-CoV-2 S-specific T cells (Table 1). These results indicated that the CD4^+^ subtypes predominantly managed the positive T cell responses to SARS-CoV-2 S protein.

**TABLE 1:** Peptides containing T cell epitopes.

	Peptide	position	Amino acid sequence	CD4 + /CD8 + T cell response	Participants
S-I-N (*N* = 2)	B38	149–163	NKSWMESEFRVYSSA	CD4	C11^a^
	B65	257–271	GWTAGAAAYYVGYLQ	CD4/CD8	C10^c^, C11^a^, C16^c^ C24^a^, C25^a^, C28^a^, C36^c^
RBD (*N* = 3)	S11	359–373	SNCVADYSVLYNSAS	CD8	C8^b^
	S14	371–385	SASFSTFKCYGVSPT	CD4	C8^a^
	S33	447–461	GNYNYLYRLFRKSNL	CD4	C28^a^
S-II (*N* = 4)	B135 B136	745–759 749–763	DSTECSNLLLQYGSF CSNLLLQYGSFCTQL	CD4 CD4	C8^a^, C11^a^ C8^a^, C11^a^
	B165	865–879	LTDEMIAQYTSALLA	CD4	C8^a^, C11^a^
	B166 B234	869–883 1141–1155	MIAQYTSALLAGTIT LQPELDSFKEELDKY	CD4 CD4/CD8	C8^a^, C11^a^ C8^c^
	B250	1205–1219	KYEQYIKWPWYIWLG	CD4/CD8	C28^c^

*^*a*^CD4^+^T cell response. ^*b*^CD8^+^T cell response. ^*C*^both CD4^+^ and CD8^+^T cell response.*

Meanwhile, we evaluate the cross-reactivity of the PBMCs from healthy people to these positive peptides *via* IFN-γ ELISA assays. Only the B250 peptide was found to induce a relatively higher level of T cell responses from 2 of 10 healthy donors (Figure 3E). Due to the reported similarity between SARS-CoV-2 and the common cold coronavirus (Mateus et al., 2020; Nelde et al., 2021), we examined the homology of the positive peptides to a few coronaviruses. We found that 10 of 11 positive peptides exhibited the highest degree of homology to SARS-CoV, followed by MERS-CoV, whereas B38 was specific to SARS-CoV-2 S protein (Figure 3F). Also, the majority of these positive peptides share a certain extent of sequence similarities to the human common cold coronaviruses 229E, HKU1, NL63, and OC43. Among them, the sequence of B250 showed a high resemblance to all coronaviruses examined. Notably, the immunodominant peptide B65 showed approximately 60% sequence similarity to SARS-CoV and 40% to MERS-CoV, while it presented relatively low homology levels to the four common cold coronaviruses (Figure 3F). These findings demonstrated the existence of SARS-CoV-2 S-reactive T cells in healthy people, and such cross-reactivity was highly likely due to the sequence similarity between the S protein and those of the human common cold coronaviruses.

### Reactivity of B65-Specific CD4^+^ TCR-T Cells to B65 Peptide *in vitro*

**FIGURE 4:**
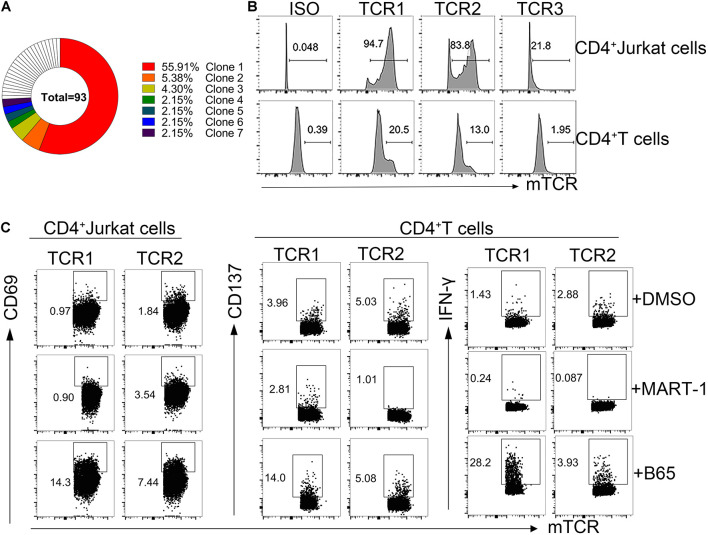
Reactivity of B65-specific CD4^+^ TCR-T cells to B65 peptide *in vitro*. **(A)** MGT/V-Quest was used to analyze the TCR repertoire of B65-specific CD4^+^ T cells. Different colors mean different TCR clones. White indicates unique clones. **(B)** The expression of TCR in CD4^+^ Jurkat or CD4^+^ T cells was detected by FC. **(C)** TCR-transduced CD4^+^ Jurkat or CD4^+^ T cells were cocultured with PBMCs plus B65 or MART-1 (negative control) for 6 or 24 h, respectively. The level of activation marker of CD69 or CD137 and IFN-γ in TCR-transduced CD4^+^ Jurkat or CD4^+^T cells was determined by FC, respectively. FC, flow cytometry; IFN-γ, interferon-γ; TCR, T cells receptors; PBMCs, peripheral blood mononuclear cells; MART-1, melanoma-associated antigen.

To identify the TCRs responsible for the specific T cell reaction to the immunodominant peptide B65, we sorted the CD134^+^CD137^+^CD4^+^ T cell population from the C11 sample at a single-cell level. All TCRs were examined by amplifying single-cell TCR genes and analyzing the TCR sequences by IMGT. Finally, a total of seven dominant TCRs were established in B65-specific TCR repertoire (Figure 4A). The top three dominant TCR clones (TCR1-3) were transduced into CD4^+^ Jurkat and the allogenic CD4^+^ T cells. As shown in Figure 4B, TCR1 and TCR2 were expressed in CD4^+^ Jurkat and CD4^+^ T cells both at high levels. To determine whether B65 could be recognized by the engineered cells, we loaded this peptide to PBMCs, which act as antigen-presenting cells, followed by a coculture with TCR-transduced CD4^+^ Jurkat or CD4^+^ T cells. The elevated expression of CD69 suggested that B65 can activate both TCR1- and TCR2-transduced Jurkat cells (Figure 4C left). Also, the increased levels of CD137 expression and IFN-γ secretion demonstrated that B65 could induce the activation of TCR1-transduced CD4^+^ T, while TCR2 expressing CD4^+^ T was not responsive (Figure 4C right). These results showed that we successfully identified a specific CD4^+^ TCR against B65. In conclusion, we presented a dominant epitope of SARS-CoV-2 S from the PBMCs of COVID-19 convalescent patients, represented by the peptide B65, together with its specific functional TCR for CD4^+^ T cells.

## Discussion

This study found that SARS-CoV-2 S-specific reactive CD4 + T cells exhibited a higher frequency than CD8^+^ T cells in recovered COVID-19 patients, with a greater number of corresponding epitopes presented. Importantly, we identified a peptide B65 within the S-I-N region containing immunodominant epitopes for CD4^+^ and CD8^+^ T cells, and it could induce a strong T cell response in convalescent PBMCs. Further, we successfully obtained the CD4^+^ TCRs recognizing B65 and confirmed that the constructed B65 TCR-T could display strong functional activity against SARS-CoV-2 S. Our findings provide vital information for understanding the cellular immunity against SARS-CoV-2 and for the development of effective strategies for COVID-19 treatment.

Compared with healthy people, COVID-19 convalescent patients were shown with similar T cells, yet they contained a significantly larger proportion of T_CM_ cells in their PBMCs. This finding was in line with the previous discovery that the majority of SARS-CoV-2-specific T cells exhibited T_CM_ phenotypes (Peng et al., 2020). Due to the limitation of sample size, we did not determine the specificity of these T_CM_ cells. Nevertheless, the overall robust response to the S protein indicated that recovered patients from COVID-19 might exhibit resistance to SARS-CoV-2-antigen reexposure. Moreover, we presented the evidence that both the frequency and epitopes of SARS-CoV-2-specific CD4^+^ T cells were higher than those of CD8^+^ T cells, which were consistent with recent findings in COVID-19 convalescents (Grifoni et al., 2020; Sekine et al., 2020). Given the divergent role of CD4^+^ T cells in regulating both humoral immunity and cellular immunity, we suspect that the relatively mild disease severity of these COVID-19 patients included in our study may benefit from a stronger CD4^+^ T cell response to SARS-CoV-2. This was supported by the recent finding that the number of SARS-CoV-2 S-specific CD4^+^ T cells was positively correlated with the anti-S RBD IgG titers and magnitude of SARS-COV-2 S-specific CD8^+^ T cells (Grifoni et al., 2020).

Knowledge obtained from studying SARS-CoV indicates that B cells and antibodies are comparatively short-lived (1–2 years), whereas memory T cells exhibit a longer life span (>6–17 years) (Channappanavar et al., 2014b; Zhao et al., 2016; Lin et al., 2020). Therefore, T cell immunity can be used to evaluate the timely effectiveness of vaccines and as a reflection of the long-term protection provided by vaccines. In this study, we evidenced that B65 within the S-I-N region of SARS-CoV-2 S could be recognized by the PBMCs of almost all participants tested, indicating that T cell responses and neutralizing antibodies might favor different regions of the S protein. To be noted, B65 does not contain any amino acids corresponding to the mutations of the highly infectious SARS-CoV-2 variants, such as B.1.1.7, B.1.351, B.1.28, and B.1.617 (Supplementary Material). Hence, B65 may serve as a valuable addition for assessing specific T cell responses post-vaccination, and it can also be used as a potential candidate for peptide vaccine development to trigger effective T cell response.

The T cell immune response plays an essential role in intracellular viral clearance (Yang et al., 2020; Weinreich et al., 2021). However, in COVID-19 patients, the total number of CD4^+^ and CD8^+^ T cells was significantly reduced, and their phenotype exhibited exhaustion markers, which usually lead to the functional impairment of T cells (Diao et al., 2020; Zheng H. Y. et al., 2020; Zheng M. et al., 2020). Hence, improving virus-specific T cell response may be the key to recovering COVID-19 patients, especially for severe cases. Adoptive T cell therapies have been proven to be an effective treatment approach against malignant tumors and viral infections (Leen et al., 2006). Studies have shown that there were SARS-CoV-2-specific memory T cells in COVID-19 convalescents (Ferreras et al., 2021; Perez-Martinez et al., 2021), and the symptoms were significantly improved in patients with SARS-CoV-2 after being treated with SARS-CoV-2-specific memory T cells (Perez-Martinez et al., 2021). This result indicated that adoptive T cell therapies are an effective treatment for COVID-19. This study presented an S-specific TCR targeting the immunodominant peptide B65, with functional significance against SARS-CoV-2. Future studies with an enhanced transfection efficiency of TCRs and corresponding detailed functional verification *in vivo* may significantly increase the efficacy of SARS-CoV-2-specific CD4^+^ TCR-T cells and help us to better evaluate this potential therapeutic method for treating COVID-19 patients.

Both TCR and HLA are the restriction factors for TCR-T therapies. Previous work identified 30 HLA class I and 45 HLA class II alleles of HLA-restricted epitopes, which cover the most common specificities in the general worldwide population (Grifoni et al., 2021). To cover most populations, more TCR should be acquired based on these epitopes. Especially, to avoid host versus graft reaction (HVGR) and graft versus host reaction (GVHR), the patient autologous T cells can be used to construct TCR-T cells when treating COVID-19.

In conclusion, we confirmed that SARS-CoV-2 S protein induces strong and broad CD4^+^ T cell responses in the majority of COVID-19 convalescent patients. Further, we presented an immunodominant epitope B65 which might be a valuable candidate for peptide vaccine development. Importantly, SARS-CoV-2-specific-TCR-T cells could be a new approach of COVID-19 treatment.

## Data Availability Statement

The original contributions presented in the study are included in the article/Supplementary Material, further inquiries can be directed to the corresponding author/s.

## Ethics Statement

The studies involving human participants were reviewed and approved by Ethics Committee of Chongqing Medical University. The patients/participants provided their written informed consent to participate in this study. Written informed consent was obtained from the individual(s) for the publication of any potentially identifiable images or data included in this article.

## Author Contributions

AJ designed the study. XH, QC, MS, CH, SC, TL, YW, YH, JH, and SL were responsible for collecting the samples. LL, XH, QC, MS, CH, SC, and JZ performed the experiments. LL, XH, and QC performed the single-cell TCR cloning. LL and QC performed the data analysis. AJ, LL, and QC wrote the manuscript. All authors contributed to the article and approved the submitted version.

## Conflict of Interest

The authors declare that the research was conducted in the absence of any commercial or financial relationships that could be construed as a potential conflict of interest.

## Publisher’s Note

All claims expressed in this article are solely those of the authors and do not necessarily represent those of their affiliated organizations, or those of the publisher, the editors and the reviewers. Any product that may be evaluated in this article, or claim that may be made by its manufacturer, is not guaranteed or endorsed by the publisher.

## Footnotes

1
https://coronavirus.jhu.edu/map.html
