# Supplementary material for: T Cell Immunity Evaluation and Immunodominant Epitope T Cell Receptor Identification of Severe Acute Respiratory Syndrome Coronavirus 2 Spike Glycoprotein in COVID-19 Convalescent Patients
Source: Front Cell Dev Biol. 2021 Nov 3;9:696662. doi: 10.3389/fcell.2021.696662 (PMC8595245; doi:10.3389/fcell.2021.696662)
Supplement: Supplementary file 1 [file Data_Sheet_1.PDF]

Supplementary: SARS-CoV-2 spike glycoproteins variants

Region	Lineage	Spike Mutation
Brazil	B.1.1.28	L18F T20N P26S D138Y R190S K417T E484K N501Y D614G H655Y T1027I V1176F
UK	B.1.1.7	69-70del 144del N501Y A570D D614G P681H T716I S982A D1118H
SA	B.1.351	L18F D80A D215G 242-244del K417N E484K N501Y D614G A701V
India	B.1.617	G142D E154K L452R E484Q D614G P681R Q1071H H1101D

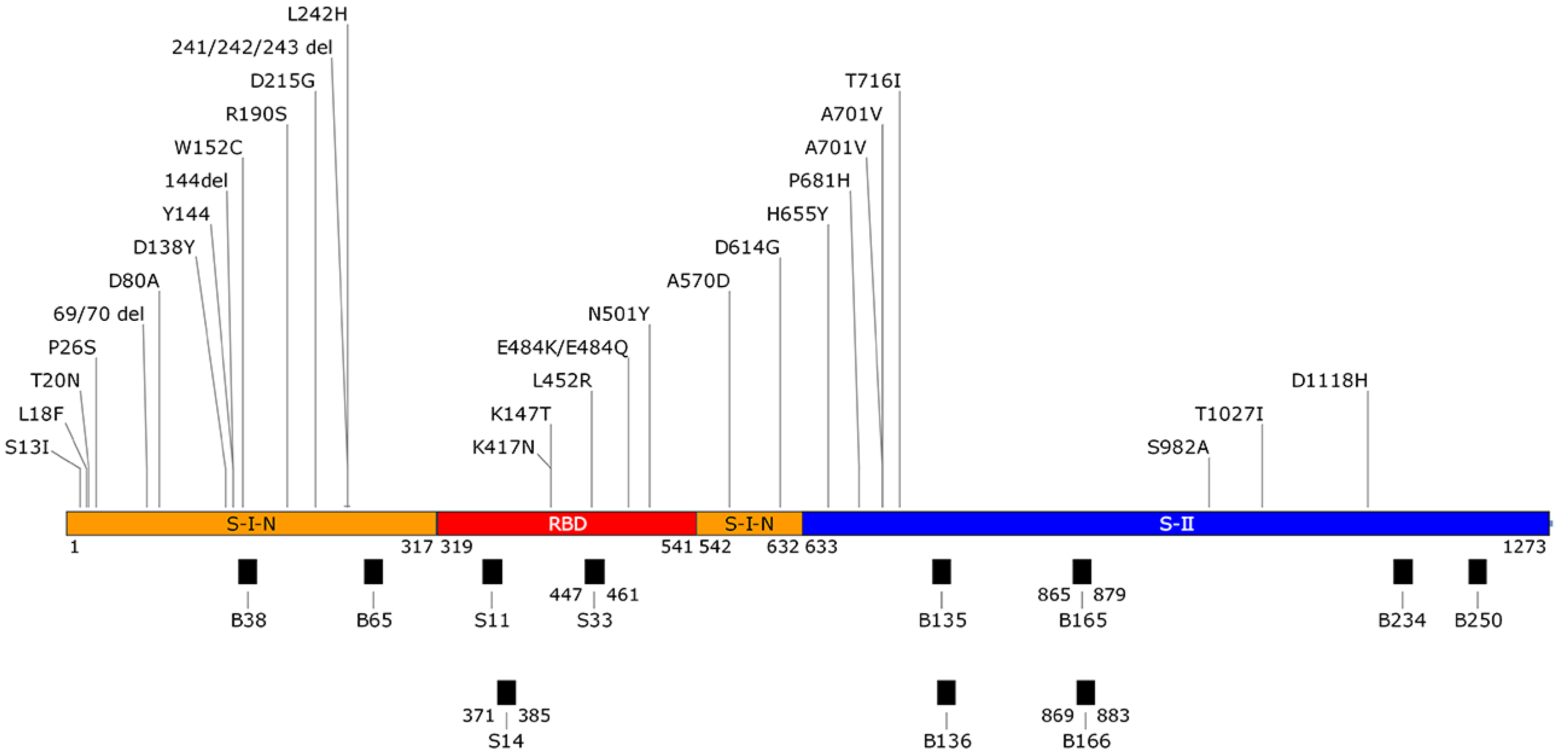

SARS-CoV-2 S Protein

1274 aa
